# Rate and nature of complications with leadless transcatheter pacemakers compared with transvenous pacemakers: results from an Italian multicentre large population analysis

**DOI:** 10.1093/europace/euac112

**Published:** 2022-08-29

**Authors:** Pietro Palmisano, Domenico Facchin, Matteo Ziacchi, Gerardo Nigro, Antonino Nicosia, Maria Grazia Bongiorni, Luca Tomasi, Andrea Rossi, Paolo De Filippo, Giuseppe Sgarito, Roberto Verlato, Michele Di Silvestro, Saverio Iacopino

**Affiliations:** Cardiology Unit, “Card. G. Panico” Hospital, Tricase, Italy; SOC Cardiologia-Dipartimento Cardiotoracico, Azienda Sanitaria Universitaria Friuli Centrale, Udine, Italy; Istituto di Cardiologia, Policlinico Sant’Orsola Malpighi, Università degli Studi di Bologna, Bologna, Italy; A.O. Monaldi, Napoli, Italy; Azienda Sanitaria Provinciale, Ospedale Giovanni Paolo II, Ragusa, Italy; Azienda Ospedaliera Universitaria Ospedale Cisanello, Pisa, Italy; Azienda Ospedaliera Universitaria, Ospedale Borgo Trento, Verona, Italy; Fondazione Toscana Gabriele Monasterio, Pisa.Italy; Ospedale Giovanni Paolo XXIII, Bergamo, Italy; Ospedale Civico, Palermo, Italy; ULSS 6 Euganea, Camposampiero-Cittadella, Cittadella, Italy; Azienda Sanitaria Provinciale, Ospedale Umberto I, Enna, Italy; Maria Cecilia Hospital, Cotignola, Italy

**Keywords:** Leadless pacemaker, Transvenous pacemaker, Complications, Lead-related complications, Pocket-related complications, Device infection

## Abstract

**Aims:**

The safety and efficacy of leadless intracardiac-permanent pacemaker (L-PM) have been demonstrated in multiple clinical trials, but data on comparisons with conventional transvenous-permanent pacemaker (T-PM) collected in a consecutive, prospective fashion are limited. The aim of this analysis was to compare the rate and the nature of device-related complications between patients undergoing L-PM vs. T-PM implantation.

**Methods and results:**

Prospective, multicentre, observational project enrolling consecutive patients who underwent L-PM or T-PM implantation. The rate and nature of device-related complications were analysed and compared between the two groups. Individual 1:1 propensity matching of baseline characteristics was performed. A total of 2669 (*n* = 665 L-PM) patients were included and followed for a median of 39 months, L-PM patients were on average older and had more co-morbidities. The risk of device-related complications at 12 months was significantly lower in the L-PM group (0.5% vs. 1.9%, *P* = 0.009). Propensity matching yielded 442 matched pairs. In the matched cohort, L-PM patients trended toward having a lower risk of overall device-related complications (*P* = 0.129), had a similar risk of early complications (≤30 days) (*P* = 1.000), and had a significantly lower risk of late complications (>30 days) (*P* = 0.031). All complications observed in L-PM group were early. Most (75.0%) of complications observed in T-PM group were lead- or pocket-related.

**Conclusion:**

In this analysis, the risk of device-related complications associated with L-PM implantation tended to be lower than that of T-PM. Specifically, the risk of early complications was similar in two types of PMs, while the risk of late complications was significantly lower for L-PM than T-PM.

**What’s new?**

- After adjustment for patient characteristics, the risk of major device-related complications associated with leadless pacemaker implantation tended to be lower than that of conventional transvenous pacemaker.
- The risk of early complication was similar in the two types of pacemaker, whereas the risk of late complications was significantly lower in leadless pacemaker than transvenous pacemaker.
- The lower risk of late complications observed in leadless pacemaker patients was mainly due to the absence of lead- and pocket-related complications, which accounted for more than 70% of all complications observed in transvenous pacemaker patients.

## Introduction

The permanent pacemaker (PM) is the cornerstone of the treatment of symptomatic and severe bradycardia due to bradyarrhythmia and conduction disorders.^1–4^ Despite the impressive technological development of implantable electronic cardiac devices, the implantation technique of a conventional transvenous-PM (T-PM) involving a subcutaneous pulse generator and transvenous leads has remained unchanged. The subcutaneous pocket and the transvenous leads are the most common source of complications related to the implantation of a T-PM, occurring in up to 3–12% of patients.^5–8^

To overcome lead- and pocket-related complications, leadless intracardiac PM (L-PM) systems have been developed as a therapeutic alternative to T-PM therapy. Leadless intracardiac PM are completely implanted in the right ventricle by means of a minimally invasive procedure through the femoral vein and are currently implanted in patients with an indication for single-chamber ventricular pacing.^9^

Currently, one L-PM is routinely used in many European centres: the Micra Transcatheter Pacing System (TPS, Medtronic, Inc., Minneapolis, MN, USA). The efficacy and safety of Micra TPS among guideline-indicated^1^ patients have been evaluated in the investigational Micra Transcatheter Pacing Study^9–11^ and confirmed in a real-world setting in the worldwide Micra TPS Post-Approval Registry (PAR).^12,13^ In these studies, this L-PM demonstrated optimal and stable pacing performance, low rate of major complications, and exhibited high implantation success rates.

In two *post hoc* analyses, the risk of device-related complications of patients enrolled in the Micra Transcatheter Pacing Study^11^ and in the Micra TPS PAR^13^ was compared with that of a predefined historical control group of patients who underwent T-PM therapy. In both studies, Micra TPS was associated with a significant reduction in the risk of major complications (of 48 and 63% at 12 months, respectively).^11,13^ Although these findings were highly significant, these studies were limited by the retrospective nature of the analysis.

Recently, in the Longitudinal Coverage With Evidence Development Study on Micra Leadless Pacemakers (Micra CED), a prospective, observational Medicare claims study, the risk of complications of patients undergoing Micra TPS implantation was compared with that of a contemporary cohort of patients undergoing VVI T-PM implantation.^14^ In this analysis, L-PM was associated with a reduction of 23% in the risk of device-related complications compared with T-PM at 6 months post-implant.

We conducted a large population, multicentre project aimed to prospectively compare the rate and nature of device-related complications between L-PM and a cohort of patients who were referred for T-PM implantation in the same centres, by the same operators and during the same period of time.

## Methods

### Study population

The present study was a prospective, multicentre, observational project involving 16 medium/high volume Italian centres participating to the One Hospital ClinicalService project, designed to prospectively collect data on complications and outcomes of patients undergoing PM implantation during long-term follow-up. One Hospital ClinicalService is a medical care project targeted to quality improvement in the use of Medtronic devices in clinical practice. This project was approved by each site’s Institutional Review Board and Local Ethics Committees. The design conforms to the principles outlined in the 1975 Declaration of Helsinki as reflected in the *a priori* approval by the institution’s human research committee. Each patient included in the One Hospital ClinicalService project provided informed consent for the data collection and analysis.

All consecutive patients undergoing *de novo* PM implantation (L-PM or T-PM) at participating centres from May 2016 through December 2019 were prospectively included in this analysis. Included patients met Class I or II guideline recommendations^1^ for permanent pacing. Owing to the increased risk of complications related to the coronary sinus lead, patients receiving a biventricular PM were excluded from the analysis. The choice of T-PM or L-PM was based on physician preference and the patients’ clinical characteristics. In general, an L-PM was preferentially implanted in patients with difficult or unavailable venous access for T-PM implantation, in patients at high risk of infection, and in patients who undergo extraction of a T-PM for infection.

### Implantation procedure

At the time of implantation, data on baseline characteristics, clinical indications, type of device implanted, and procedural details (including intra-procedural complications) were collected.

All PM implantation procedures were performed in the electrophysiology laboratory under local anaesthesia by electrophysiologists experienced in device therapy. All operators who performed L-PM implantation procedures were properly trained in this technique. Prophylactic antibiotic treatment was administered perioperatively in all procedures according to local protocols.

Peri-procedural oral anticoagulant therapy management was similar in all participating centres and in both L-PM and T-PM implantation procedures. Vitamin K antagonists were not discontinued and the international normalized ratio was checked on the day of the procedure (not >2.5). Direct oral anticoagulants were discontinued at least 24 h prior to the procedure and were restarted from 12 to 24 h after the procedure.

#### Leadless intracardiac-permanent pacemaker implantation procedure

In the study period, the L-PM implanted at participating centres was the Micra TPS. The technical characteristics of the device and the implant procedure have been described previously.^9,10,15,16^ The device was primarily intended to be implanted in the right ventricular (RV) septo-apical region; if this was not accessible, it was implanted in any other position that resulted in good pacing and detection parameters. Each centre utilized their own standard-of-care practices and approaches during the implant.

#### Conventional transvenous-permanent pacemaker implantation procedure

Venous access for the RV and the atrial leads (for dual-chamber T-PMs) was achieved by direct puncture of the subclavian or axillary vein or by surgical isolation of the cephalic vein. The RV lead was preferentially positioned in the RV septo-apical region if possible, or in the RV apex if septal positioning failed. The atrial lead was preferentially positioned in the right atrial appendage. The pulse generator was routinely placed subcutaneously.

### Data collection and follow-up

The patients were examined 1 week after discharge and then at 6–12-month intervals, according to each centre’s routine practice. At each scheduled or unscheduled outpatient clinic visit, the pocket (in T-PM patients) or the femoral venous access site (in L-PM patients) was assessed, the device was interrogated, and the integrity and appropriate functioning of the pacing system were checked.

When a predefined device-related complication (see below) was observed, all information (clinical data, device interrogations, and surgical reports) was carefully collected and analyzed.

### Definitions of complications

The primary outcome of the study was device-related complications. Device-related complications were predefined as any device-related adverse event that was identified after the implantation procedure resulting in death, permanent loss of device function due to mechanical or electrical dysfunction, hospitalization, prolonged hospitalization by at least 48 h, or pacing system surgical revision.^7,8,11^

Device-related adverse events included cardiac tamponade, device infection (systemic or local infection), device malfunction, pneumothorax, pocket haematoma, lead dislodgement, lead failure, femoral access-site complication, and device migration. Cardiac tamponade was defined as pericardial effusion causing hemodynamic compromise and requiring drainage. Device infection was classified as either systemic device-related infection or local infection. Systemic infection requiring complete removal of the pacing system was confirmed when valvular, lead, or device vegetations were detected through echocardiography or the modified Duke criteria^17^ for infective endocarditis. Local infection was defined as swelling, redness, and/or discharge in the pocket region or at the femoral venous access-site along with bacterial growth in wound cultures, without evidence of systemic infection. Device malfunction was defined as any dysfunction of the pulse generator irresolvable by reprogramming requiring premature replacement or deactivation. Pneumothorax was defined as the absence of lung markings over the lung X-ray field ipsilateral to the device pocket and requiring pleural drainage. Pocket haematoma requiring surgical drainage was defined as a palpable mass that protruded >2 cm associated with the presence of tense swelling or causing severe pain. Lead dislodgement was defined as inadequate capture or sensing irresolvable by device reprogramming associated with a visible change in the lead device position on chest X-ray and requiring surgical revision.^7^ Lead failure was defined as a sudden non-physiological change in impedance associated with changes in the sensing or pacing performance and without evidence of lead dislodgement.^7^ Femoral vascular access-site complications included pseudoaneurysm, arteriovenous fistula, haematoma requiring transfusion, retroperitoneal haemorrhage, embolism, thrombosis, and vessel rupture/perforation.^9^ Device migration was defined as a visible change in the device position on chest X-ray requiring retrieving or deactivation and the implantation of a new device.^9^

Conventional transvenous-PM-specific complications were defined as all pocket- and lead-related complications that are not associated with implantation of an L-PM; these included pneumothorax, pocket haematoma, lead dislodgement, and lead failure. Conversely, L-PM-specific complications were defined as all complications that are not associated with implantation of a T-PM; these included femoral vascular access-site complications and device migration.

Early complications were defined as any complication occurring during the first 30 days after PM implantation, including perioperative events. Complications that occurred after the first month post-implantation were defined as late complications.^8^

Upgrades of the pacing system to dual-chamber pacing, implantable cardioverter defibrillator, or resynchronization therapy devices were not considered device-related complications unless the upgrade was due to a device-related adverse event.

All complications reported were adjudicated by the clinical events committee. If a patient had more than one event, the complications were carefully distinguished and monitored over time. If the investigators reported events that were not pre-specified, the same committee considered each event separately, assessing its relationship to the implantation procedure.

### Statistical analysis

Descriptive statistics were reported as means and standard deviations for normally distributed continuous variables. Continuous variables with skewed distribution were reported as medians and 25–75th percentiles. The Student’s *t*-test or the Mann–Whitney *U* test was used to compare continuous variables between groups. Categorical variables were reported as percentages and compared using the χ2 test or Fisher’s exact test, as appropriate. Event and event-free curves were based on Kaplan–Meier analyses, stratified by the study group and compared using the log-rank test. Predictors of the first long-term event were analysed with a Cox regression model, and the related hazard ratios (HRs) together with their 95% confidence interval (CI) were reported. To account for important patient differences, a propensity score was computed for all eligible participants undergoing PM implantation using binary logistic regression: PM type (L-PM or T-PM) was the binary outcome and all baseline variables (*Table 1*) were used as covariates for estimating the probability (i.e. propensity score). Then, patients were matched 1:1 at the 5th digit of the propensity score: those left out were matched at the 4th digit and so forth to the 2nd digit. If an L-PM patient could not be matched to any T-PM subject on the second digit of the propensity score, then the L-PM subject was discarded from the matched analysis. All statistical tests were based on a two-sided significance level of 0.05. SAS software, version 9.4, (SAS Institute Inc., Cary, NC, USA) was used to perform all statistical analyses.

**Table 1:** Baseline characteristics of the overall unmatched population

Characteristics	Overall study population (*n* = 2669)	L-PM (*n* = 665)	T-PM (*n* = 2004)	*P*-value
*Baseline characteristics*				
ȃMale, *n* (%)	1686 (63.2)	462 (69.5)	1224 (61.1)	<0.001
ȃAge in years, mean ± SD	72.8 ± 13.4	73.9 ± 13.8	72.5 ± 13.3	0.013
ȃBody mass index (kg/m^2^)	26.8 ± 4.6	26.9 ± 4.7	26.8 ± 4.6	0.655
ȃLVEF in %, mean ± SD	56.2 ± 8.2	55.6 ± 8.1	58.4 ± 8.5	<0.001
ȃLVEF < 50%, *n* (%)	338 (12.7)	89 (13.4)	249 (12.4)	0.427
ȃNYHA Class > II, *n* (%)	185 (6.9)	67 (10.2)	118 (5.9)	0.023
*Associated disorders*				
ȃHypertension on therapy, *n* (%)	1748 (65.5)	496 (74.7)	1252 (62.5)	<0.001
ȃDiabetes, *n* (%)	576 (21.6)	163 (24.4)	413 (20.6)	0.069
ȃLeft bundle-branch block, *n* (%)	160 (6.0)	43 (6.4)	116 (5.8)	0.595
ȃCongestive heart failure, *n* (%)	411 (15.4)	119 (17.9)	292 (14.6)	0.072
ȃIschemic cardiopathy, *n* (%)	96 (3.6)	56 (8.4)	40 (2.0)	<0.001
ȃAtrial fibrillation, *n* (%)	1315 (49.2)	445 (67.0)	870 (43.4)	<0.001
ȃHistory of TIA/stroke, *n* (%)	218 (8.2)	62 (9.5)	156 (7.8)	0.245
ȃRenal dysfunction, *n* (%)	366 (13.7)	133 (20.4)	233 (11.7)	<0.001
ȃChronic obstructive pulmonary disease, *n* (%)	283 (10.6)	115 (17.4)	168 (8.4)	<0.001
*Cardiovascular medications*				
ȃOral anticoagulant, *n* (%)	1170 (43.8)	416 (62.6)	754 (37.6)	<0.001
ȃDiuretics, *n* (%)	976 (36.6)	313 (47.1)	663 (33.1)	<0.001
ȃAntiplatelets, *n* (%)	834 (31.2)	151 (22.7)	683 (34.1)	<0.001
ȃACE inhibitors, *n* (%)	784 (29.4)	191 (28.7)	593 (29.6)	0.670
ȃBeta-blockers, *n* (%)	744 (27.9)	170 (25.6)	574 (28.6)	0.125
ȃARBs, *n* (%)	398 (14.9)	85 (12.8)	313 (15.6)	0.075
ȃDigoxin, *n* (%)	129 (4.8)	27 (4.1)	102 (5.1)	0.283
ȃMRAs, *n* (%)	89 (3.3)	25 (3.8)	64 (3.2)	0.481
ȃIvabradine, *n* (%)	37 (1.4)	19 (2.9)	18 (0.9)	<0.001
ȃARNi, *n* (%)	3 (0.1)	1 (0.2)	2 (0.1)	0.736

ACE, angiotensin-converting enzyme; ARB, angiotensin receptor blocker; ARNi, angiotensin receptor-neprilysin inhibitor; L-PM, leadless intracardiac pacemaker; LVEF, left ventricular ejection fraction; MRA, mineralocorticoid receptor antagonist; NYHA, New York Heart Association; SD, standard deviation; TIA, transient ischaemic attack; T-PM, conventional transvenous pacemaker.

## Results

### Overall study population

A total of 2669 consecutive patients undergoing PM implantation from May 2016 through December 2019 were included in the analysis, 665 undergoing L-PM implantation and 2004 undergoing T-PM implantation. In all participating centres, both types of PM were implanted during the study period. The rate of L-PMs out of the total number of PMs included in the analysis was different among the 16 participating centres, specifically, was more than 50% in three centres, from 10–50% in three centres, and less than 10% in the remaining 10 centres.

L-PM patients were more often male, were older, and had more co-morbidities than T-PM patients (*Table 1*). Among the 2004T-PMs implanted, 102 (5.1%) were single-chamber and 1902 (94.9%) were dual-chamber. The median duration of follow-up for the whole population was 39 (interquartile range: 16–83) months and no patients were lost to follow-up.

Pacing thresholds were low and remained stable during follow-up for L-PM (*Table 2*). Pacing thresholds increased significantly over follow-up for T-PM, although the percent of patients with thresholds >1 V was similar for both groups. Impedance and sensing were stable for both groups; however, the proportion of patients with sensing <5 mV was significantly higher for T-PM.

**Table 2:** Electrical parameters at implantation and at 1-year follow-up, comparison between L-PM and T-PM

Characteristics	At implant			1-year follow-up			P-value for L-PM at implant vs. L-PM at follow-up	P-value for T-PM at implant vs. T-PM at follow-up
	L-PM (*n* = 665)	T-PM (*n* = 2004)	P-value for L-PM vs. T-PM	L-PM (*n* = 665)	T-PM (*n* = 2004)	P-value for L-PM vs. T-PM		
Impedance in Ohm, mean ± SD	739.4 ± 169.9	672.0 ± 196.5	<0.001	557.2 ± 102.4	534.7 ± 97.1	0.002	<0.001	<0.001
Sensing in mV, mean ± SD	10.5 ± 4.8	10.1 ± 5.0	0.109	11.1 ± 4.8	9.7 ± 5.0	<0.001	<0.001	<0.001
Patients with sensing <5 mV, *n* (%)	63 (9.5%)	300 (15.0%)	0.002	60 (9.0%)	340 (17.0%)	<0.001	0.505	0.011
Ventricular pacing threshold amplitude, in V at 0.24 ms for L-PM, in V at 0.5 ms for T-PM, mean ± SD	0.5 ± 0.3	0.6 ± 0.3	<0.001	0.6 ± 0.3	0.8 ± 0.3	<0.001	0.057	<0.001
Patients with ventricular pacing threshold amplitude >1 V, *n* (%)	45 (6.8%)	122 (6.1%)	0.615	53 (8.1%)	178 (8.9%)	0.746	1.000	0.012

L-PM, leadless intracardiac pacemaker; SD, standard deviation; T-PM, conventional transvenous pacemaker.

During follow-up device-related complications were observed in three patients in the L-PM group (0.5; 95% CI: 0.1–1.4%), and in 45 patients in T-PM group (2.3; 95% CI: 1.6–2.0%; *P* = 0.003 compared with L-PM group). There is not a significant difference in complication rate amongst the participating centres (median: 0.0%, 25–75th percentiles: 0.0–2.4; *P* = 0.205). Arteriovenous fistula at the femoral vascular access-site occurred in one L-PM patient and was treated with surgery within 24 h of the implantation procedure. The second L-PM complication was a device malfunction with total loss of capture ∼24 h after the implantation procedure; the device was inactivated and abandoned, and a new T-PM was implanted. The third L-PM complication was a device migration observed in one patient 48 h after the implantation procedure; the device was retrieved without complications, and a new L-PM was implanted.

Thirty-four of 45 complications observed in T-PM group (75.6%) were T-PM-specific as they were lead- or pocket-related (*Table 3*, left columns)

**Table 3:** Rate and nature of device-related complications observed during the study period in general population and in propensity score matched cohort

Characteristics	General population			Propensity score matched		
	L-PM (*n* = 665)	T-PM (*n* = 2004)	*P*-value	L-PM (*n* = 442)	T-PM (*n* = 442)	*P*-value
Patients with at least one complication, *n* (%)	3 (0.5)	45 (2.3)	0.003	3 (0.7)	8 (1.8)	0.129
ȃEarly complications, *n* (%)	3 (0.5)	9 (0.5)	0.995	3 (0.7)	2 (0.5)	0.654
ȃLate complications, *n* (%)	0 (0.0)	36 (1.8)	<0.001	0 (0.0)	6 (1.4)	0.014
*General complications*						
ȃPericardial effusion/Cardiac tamponade, *n* (%)	0 (0)	0 (0)	1.000	0 (0)	0 (0)	1.000
ȃDevice infection, *n* (%)	0 (0)	10 (0.5)	0.068	0 (0)	2 (0.5)	0.499
ȃSystemic infection, *n* (%)	0 (0)	4 (0.2)	0.249	0 (0)	1 (0.2)	1.000
ȃLocal infection, *n* (%)	0 (0)	6 (0.3)	0.158	0 (0)	1 (0.2)	1.000
ȃDevice malfunction, *n* (%)	1 (0.2)	0 (0)	0.083	1 (0.2)	0 (0)	1.000
ȃPremature battery depletion *n* (%)	0 (0)	1 (0.0)	1.000	0 (0)	0 (0)	1.000
*T-PM-specific complications*						
ȃPneumothorax, *n* (%)	—	0 (0)	—	—	0 (0)	—
ȃPocket haematoma, *n* (%)	—	7 (0.4)	—	—	2 (0.5)	—
ȃLead dislodgement, *n* (%)	—	24 (1.2)	—	—	4 (0.9)	—
ȃAtrial lead, *n* (%)	—	11 (0.6)	—	—	1 (0.2)	—
ȃRV lead, *n* (%)	—	13 (0.7)	—	—	3 (0.6)	—
ȃLead failure, *n* (%)	—	3 (0.2)	—	—	0 (0)	—
ȃAtrial lead, *n* (%)	—	2 (0.1)	—	—	0 (0)	—
ȃRV lead, *n* (%)	—	1 (0.0)	—	—	0 (0)	—
**L-PM-specific complications**						
ȃFemoral vascular access—site complications, *n* (%)	1 (0.2)	—	—	1 (0.2)	—	—
ȃDevice migration, *n* (%)	1 (0.2)	—	—	1 (0.2)	—	—

L-PM, leadless intracardiac pacemaker; RV, right ventricular; T-PM, conventional transvenous pacemaker.

**Figure 1:**
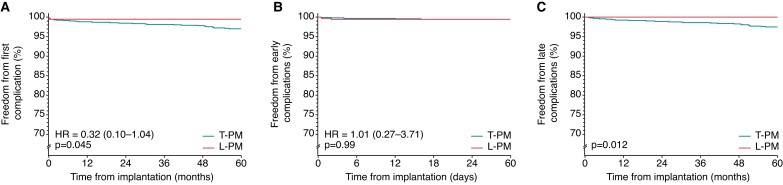
*Panel A*: Freedom from first device-related complication in overall study population, comparison between L-PM and T-PM patients. *Panel B*: Freedom from early device-related complications in L-PM and T-PM groups. Panel C: Freedom from late device-related complications in L-PM and T-PM groups. HR, hazard ratio; L-PM, leadless intracardiac pacemaker; T-PM, conventional transvenous pacemaker.

Compared with T-PM patients, L-PM patients had a significantly lower rate of device-related complications (0.5% vs. 2.3%, *P* = 0.003; *Table 3*, left columns). The 60-months Kaplan–Meier survival analysis showed a 68% relative risk reduction (OR: 0.32; 95% CI, 0.10–1.04; *P* = 0.045) of device-related complications in L-PM patients compared with T-PM group (*Figure 1*, panel A). Excluding the complications related to atrial lead observed in the T-PM group, the rate of device-related complications of L-PM group remained significantly lower compared with those of T-PM group (0.5% vs. 1.6%; *P* = 0.024). Compared with T-PM patients, L-PM patients had a similar risk of early complications (0.5% vs. 0.5%; *P* = 0.995), a significantly lower risk of late complications (0% vs. 1.8%; *P* < 0.001), and a trend towards lower risk of device infection (0% vs. 0.5%; *P* = 0.068; *Table 3*, left columns). The 60-months Kaplan–Meier survival analysis confirmed a similar cumulative risk of early complications in both groups (*P* = 0.990; *Figure 1*, panel B) and a significantly lower cumulative risk of late complications in L-PM patients compared with T-PM patients (*P* = 0.012; *Figure 1*, panel C).

When L-PM patients were compared with the subgroup of single-chamber T-PM patients (*n* = 102), they showed a significantly lower risk of overall device-related complications (0.5% vs. 4.0%; *P* = 0.008), a similar risk of early complications (0.5% vs. 1.0%; *P* = 0.437), and a significantly lower risk of late complications (0% vs. 3.0%; *P* = 0.002) (see Supplementary material online, Table S1).

**Figure 2:**
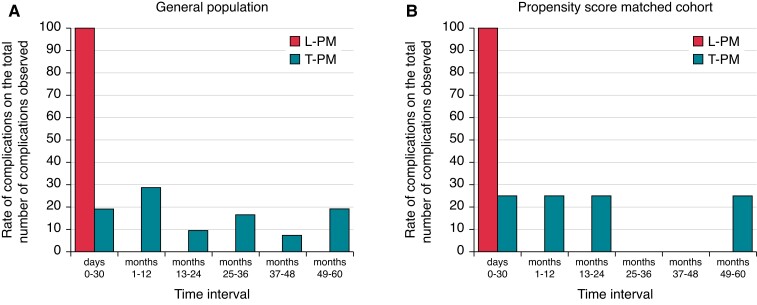
*Panel A*: Incidence of device-related complications during follow-up in overall study population, comparison between L-PM and T-PM patients. *Panel B*: Incidence of device-related complications during follow-up in L-PM and T-PM matched groups. L-PM, leadless intracardiac pacemaker; T-PM, conventional transvenous pacemaker.

As shown in *Figure 2*, panel A, all device-related complications observed in L-PM group were early, occurring in the first 30 days of follow-up. Specifically, all complications occurred within 48 h of the implantation procedure. Complications observed in the T-PM group had the highest incidence in the first year of follow-up. In subsequent years, a substantially homogeneous incidence was observed, with a slight increase observed in the latest durations of follow-up (*Figure 2*, panel A, grey bars).

### Matched cohort

Propensity score matching identified 442 pairs of patients with balanced baseline characteristics and no significant differences, which were used for analysis (*Table 4*). Nineteen T-PM patients (4.3%) included in the matched cohort received a single-chamber T-PM.

**Table 4:** Baseline characteristics of the propensity score matched cohort

Characteristics	L-PM (*n* = 442)	T-PM (*n* = 442)	*P*-value
*Baseline characteristics*			
ȃMale, *n* (%)	302 (68.3)	304 (68.8)	0.885
ȃAge in years, mean ± SD	73.2 ± 15.3	74.0 ± 12.6	0.543
ȃBody mass index (kg/m^2^)	26.9 ± 4.7	26.8 ± 4.6	0.477
ȃLVEF in %, mean ± SD	55.3 ± 8.5	55.5 ± 8.8	0.642
ȃLVEF < 50%, *n* (%)	63 (14.3)	70 (15.8)	0.736
ȃNYHA class > II, *n* (%)	41 (9.3)	34 (7.7)	0.398
*Associated disorders*			
ȃHypertension on therapy, *n* (%)	325 (73.5)	331 (74.9)	0.645
ȃDiabetes, *n* (%)	108 (24.4)	101 (22.9)	0.615
ȃLeft bundle-branch block, *n* (%)	33 (7.5)	33 (7.5)	1.000
ȃCongestive heart failure, *n* (%)	79 (17.9)	64 (14.5)	0.171
ȃIschemic cardiopathy, *n* (%)	35 (7.9)	25 (5.7)	0.181
ȃAtrial fibrillation, *n* (%)	17 (4.2)	18 (4.4)	0.889
ȃHistory of TIA/stroke, *n* (%)	32 (7.2)	37 (8.3)	0.551
ȃRenal dysfunction, *n* (%)	77 (17.4)	84 (19.0)	0.542
ȃChronic obstructive pulmonary disease, *n* (%)	58 (13.1)	44 (10.0)	0.141

L-PM, leadless intracardiac pacemaker; LVEF, left ventricular ejection fraction; NYHA, New York Heart Association; SD, standard deviation; TIA, transient ischaemic attack; T-PM, conventional transvenous pacemaker.

**Figure 3:**
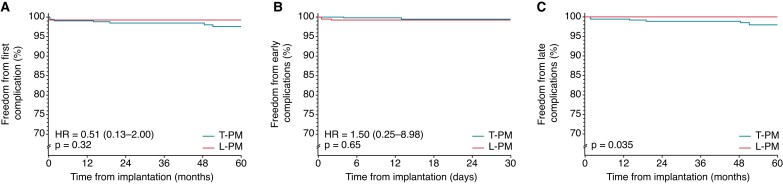
*Panel A*: Freedom from first device-related complication in L-PM and T-PM matched groups. *Panel B*: Freedom from early complications in L-PM and T-PM matched groups. *Panel C*: Freedom from late complications in L-PM and T-PM matched groups. HR, hazard ratio; L-PM, leadless intracardiac pacemaker; T-PM, conventional transvenous pacemaker.

Compared with T-PM, L-PM patients showed a similar risk of overall device-related complications (0.7% vs.1.8%; *P* = 0.129). Most of the complications observed in T-PM group (75.0%) were T-PM-specific being lead- or pocket-related (*Table 3*, right columns). In one case, the lead-related complications observed in T-PM matched group involved the atrial lead (*Table 3*, right columns). Compared with T-PM patients, L-PM patients showed a similar risk of early complications (0.7% vs. 0.5%; *P* = 0.654) and a significantly lower risk of late complications (0% vs. 1.4%; *P* = 0.013; *Table 3*, the right columns). The 60-month Kaplan–Meier survival analysis showed a similar cumulative risk of overall device-related complications (*P* = 0.320; *Figure 3*, panel A), and of early complications (*P* = 0.650; *Figure 3*, panel B) in both groups, and a significantly lower cumulative risk of late complications in L-PM patients compared with T-PM patients (*P* = 0.035; *Figure 3*, panel C).

As observed in the overall study population, also in the matched cohort all device-related complications observed in L-PM group occurred in the first month of follow-up. Conversely, in the T-PM group, complications occurred throughout the duration of follow-up, with a maximum incidence in the first year (*Figure 2*, panel B).

### Discussion

We report one of the largest prospective, multicentre, large population analyses aimed to compare the risk of device-related complications in a population of consecutive patients undergoing L-PM implantation with that of a contemporary matched cohort of patients receiving T-PM during long-term follow-up. The primary findings of the study are as follows: (i) the risk of major device-related complications after L-PM implantation is very low; (ii) the low risk of events observed in patients receiving a L-PM is mainly due to the absence of lead- and pocket-related complications, which account for over 70% of all complications observed in patients undergoing T-PM implantation; (iii) the complications related to the implantation of an L-PM are mainly intra-procedural, as demonstrated by the observation that no late complications occurred in our cohort; and (iv) acute and long-term data confirm that L-PM show optimal and stable pacing performance over time.

Leadless-PM have been developed to overcome lead-related complications (pneumothorax, dislodgement, lead fracture, or insulation failure) and pocket-related complications (haematoma, pocket erosion, and infection) which account for more than 70% of all complications observed after implantation of a T-PM.^7,8^

In the Micra Transcatheter Pacing Study,^9,11^ a pivotal, prospective, multicentre study without consecutive controls, L-PM implantation was associated with a low rate of major complications (4.0% at 12 months) and with a high implantation success rate (99.2%). The Micra TPS PAR^13^ was a worldwide large population single-arm registry designed to assess the safety and effectiveness of the L-PM in the real-world setting. The results of this study confirmed the findings of the pivotal IDE study, showing a low rate of major complications (2.7% at 12 months) and a high rate (99.6%) of implant success.

The rates of complications of patients enrolled in the Micra IDE Study and in the Micra PAR were compared with that of a historical control cohort of 2667 patients undergoing T-PM implantation.^11,13^ These analyses provided consistent results, showing that L-PMs are associated with a significant reduction in the risk of long-term device-related complications compared with T-PM (48% and 63%, respectively).^11,13^ Data on comparisons between L-PM and T-PM collected in a prospective fashion are limited.^15,18^

Recently, the results of Micra CED were published.^14^ This was a continuously enrolling observational cohort study evaluating complications, utilization, and outcomes of leadless VVI PMs in the US Medicare fee-for-service population. In this study, the complication rates of 3,726 patients treated with Micra leadless VVI PMs were compared with those of a contemporary cohort 7265 patients treated with transvenous VVI PMs. After adjustment for patient characteristics, the rate of overall early complications (within 30 days) was similar in both groups (7.7 vs. 7.4%; *P* = 0.49); while the risk of pericardial effusion and/or perforation was significantly higher among patients with L-PMs compared with patients with T-PMs (0.8% vs. 0.4%; *P* = 0.004). Patients with L-PMs showed a reduction of 23% in the risk of 6-month complications compared with patients with T-PMs (*P* = 0.02).

Consistent with the findings of the aforementioned studies, in our analysis L-PM implantation was associated with a 68% reduction in the risk of complications compared with T-PM. In the matched cohort, the reduction in the risk of overall complications did not reach statistical significance most likely because propensity score matching led to a reduction in sample size of more than 70% and because the number of events observed during follow-up was low. The lower complication rate observed in patients receiving L-PM was mainly due to the absence of lead- and pocket-related complications, which, on the other hand, were the main source of complications in patients undergoing T-PM implantation.

In our cohort, we observed complication rates in both L-PM and T-PM patients (of 0.5% and 2.3%, respectively) significantly lower than those reported in previous studies.^11,13,14^ The range of device-related complication rates reported in literature is very wide. This variability is due to several factors, including variations in the study design, different definitions of the complications, baseline patient’s characteristics, duration of the follow-up, operator experience, and hospital implantation volume.^7,8^

Compared with the Micra CED registry, in our patients, we observed a lower rate of overall complications and a lower risk of pericardial effusion/cardiac tamponade in both L-PM and T-PM patients; specifically, we did not report any cases of pericardial effusion/cardiac tamponade in either group. This finding is likely due to the significant differences in the baseline characteristics of the enrolled patients. The patients included in our analysis were younger than those enrolled in the Micra CED registry (72.8 vs. 81.0 years) and have a lower comorbidity burden (lower prevalence of heart failure, renal dysfunction, chronic obstructive pulmonary disease, coronary artery disease, and atrial fibrillation). It is well established that advanced age and co-morbidities are the primary patient-related predisposing factors for complications.^7,8^

To exclude adverse events without significant clinical impact and those not directly related to the device, in our registry, we chose *a priori* to analyse only the complications directly related to PM implantation resulting in re-hospitalization, prolonged hospitalization by at least 48 h, and/or in pacing system surgical revision. The Micra CED registry reported results based on administrative claims data. Administrative claims data lack qualitative details related to complication complexity or severity, and it is possible that the clinical impact of adverse events could have been inadequately documented.^14^ For this reason, it is possible that in the CED the rate of some complications has been overestimated.

Although numerically different, in agreement with the results of the Micra CED registry,^14^ in our study L-PM implantation was associated with a risk of early complications similar to that of T-PM, whereas it was associated with a significant reduction in the risk of late complications. Specifically, all complications observed in L-PM patients occurred within 48 h of the implantation procedure and no other complications were observed during follow-up. Because of the characteristics and the implantation technique of this device, the possible complications related to the implantation of an L-PM are almost exclusively early and specifically intra-procedural or immediately post-procedural (cardiac perforation, vascular complications, device migration), whereas the risk of late complications (infection, device malfunction) is very low.^11,13^ Conversely, after the implantation of a T-PM, late complications, which are mainly lead- and pocket-related, are most common compared with intra-procedural complications, and their incidence tends to increase over time during follow-up.^19,20^ It is therefore possible to hypothesize that with the prolongation of the follow-up the number of late complications (mostly those with a time-dependent risk such as lead failure) would continue to increase over time in T-PM patients, while remaining substantially stable in L-PM patients.

### Study limitations

Since the baseline characteristics of the two study groups were significantly different, we utilized propensity score matching to balance the cohorts; however, this resulted in discarding 77% of the original sample, thus leading to a decrease of statistical precision in results. The present study spanned over a time frame of 39 months (median follow-up) and therefore additional unforeseen events may develop over a longer time frame, thus influencing the magnitude of complication differences over a longer follow-up.

Although there were no differences in baseline characteristics in the matched cohort, we cannot exclude residual confounding of unmeasured variables due to the observational nature of the study. A large proportion of T-PM patients were implanted with dual-chamber T-PMs, which have been associated with a higher rate of complications during short-term follow-up.^7,8,20^ However, only one complication related to the atrial lead was observed in the T-PM group of the matched cohort; therefore, the inclusion of patients with dual-chamber T-PMs should not have resulted in a significant overestimation of the risk of complications in this group.

In this analysis, we do not report any minor complication not requiring hospitalization and not resulting in a significant prolongation of hospitalization. This approach could underestimate some events including asymptomatic pericardial effusion, minor injuries at the groin site, capture threshold increase requiring only PM reprogramming.

As One Hospital ClinicalService project included only Medtronic devices, the rate of L-PM implants reported in this analysis did not reflect the actual rate of L-PMs out of the total number of PMs implanted in the participating centres during the study period.

## Conclusions

In summary, the results of this multicentre, large population, prospective analysis from a real-world setting showed that, after adjustment for patient characteristics, the risk of major device-related complications associated with L-PM implantation tended to be lower than that of T-PM. Specifically, the risk of early complication was similar in the two types of PMs, while the risk of late complications was significantly lower in L-PM than T-PM. The lower risk of late complications observed in L-PM patients was mainly due to the absence of lead- and pocket-related complications, which accounted for over 70% of all complications observed in T-PM patients.

These findings confirm the favourable efficacy and safety profile of L-PM and reinforce the idea that the future direction of cardiac pacing is leadless technology.

## Supplementary Material

euac112_Supplementary_Data

## Data Availability

The data underlying this article will be shared on reasonable request to the corresponding author.
