# Supplementary material for: Rate and nature of complications with leadless transcatheter pacemakers compared with transvenous pacemakers: results from an Italian multicentre large population analysis
Source: Europace. 2022 Aug 29;25(1):112–20. doi: 10.1093/europace/euac112 (PMC10103553; doi:10.1093/europace/euac112)
Supplement: euac112_Supplementary_Data [file euac112_supplementary_data.docx]

**Supplementary material**

**Table. Rate and nature of device-related complications observed during the study period in general population: comparison between leadless pacemaker and transvenous single-chamber pacemaker.**

| **Characteristics** | **General Population** | | |
| --- | --- | --- | --- |
|  | **L-PM (n=665)** | **Single-chamber T-PM (n=102)** | **P value** |
| Patients with at least one complication, n (%) | 3 (0.5) | 4 (4.0) | 0.008 |
| Early complications, n (%) | 3 (0.5) | 1 (1.0) | 0.437 |
| Late complications, n (%) | 0 (0.0) | 3 (3.0) | 0.002 |
| **General complications** |  |  |  |
| Pericardial effusion/Cardiac tamponade , n (%) | 0 (0) | 0 (0) | 1.000 |
| Device infection, n (%) | 0 (0) | 2 (2.0) | 0.018 |
| Systemic infection, n (%) | 0 (0) | 1 (1.0) | 0.133 |
| Local infection, n (%) | 0 (0) | 1 (1.0) | 0.133 |
| Device malfunction, n (%) | 1 (0.2) | 0 (0) | 1.000 |
| Premature battery depletion n (%) | 0 (0) | 1 (1.0) | 0.133 |
| **T-PM-specific complications** |  |  |  |
| Pneumotorax, n (%) | - | 0 (0) | - |
| Pocket hematoma, n (%) | - | 0 (0) | - |
| Lead dislodgement, n (%) | - | 1 (1.0) | - |
| Atrial lead, n (%) | - | 0 (0) | - |
| RV lead, n (%) | - | 1 (1.0) | - |
| Lead failure, n (%) | - | 0 (0) | - |
| Atrial lead, n (%) | - | 0 (0) | - |
| RV lead, n (%) | - | 0 (0) | - |
| **L-PM-specific complications** |  |  |  |
| Femoral vascular access-site complications, n (%) | 1 (0.2) | - | - |
| Device migration, n (%) | 1 (0.2) | - | - |

L-PM: leadless intracardiac pacemaker; PM: pacemaker; T: transvenous; T-PM: conventional transvenous pacemaker.
